# Phosphatidylethanol vs Transdermal Alcohol Monitoring for Detecting Alcohol Consumption Among Adults

**DOI:** 10.1001/jamanetworkopen.2023.33182

**Published:** 2023-09-12

**Authors:** Judith A. Hahn, Robin Fatch, Nancy P. Barnett, Gregory M. Marcus

**Affiliations:** 1 Department of Medicine, University of California, San Francisco; 2 Department of Behavioral and Social Sciences and the Center for Alcohol and Addiction Studies, Brown University, Providence, Rhode Island

## Abstract

**Importance:**

Alcohol biomarkers can improve detection of heavy alcohol use in clinical care, yet cutoffs for phosphatidylethanol (PEth), a blood biomarker, have not been established.

**Objective:**

To determine the optimal cutoff for PEth for heavy alcohol consumption in a study of middle-age and older adults.

**Design, Setting, and Participants:**

This was a 4-week diagnostic study of adults with paroxysmal atrial fibrillation (AF) and current alcohol consumption, recruited from general cardiology and cardiac electrophysiology outpatient clinics from September 2014 to September 2019. Data were analyzed from October 2021 to March 2022.

**Main Outcomes and Measures:**

The main aim was to determine the optimal PEth cutoff for heavy alcohol consumption, using the Secure Continuous Remote Alcohol Monitor (SCRAM) to measure transdermal alcohol. Area under the curve (AUC) for PEth-detected compared with SCRAM-detected heavy alcohol consumption in any week over the prior 4 weeks (ie, ≥3 [women] and ≥4 [men] episodes) or any estimated breath alcohol of 0.08% or greater in any week, and the PEth cutoff was calculated using the Youden J statistic. Similar analyses were conducted comparing PEth with individual drinks reported by pressing an event monitor, retrospective self-report via the Alcohol Use Disorders Identification Test–Consumption (AUDIT-C), and using 2-week look-backs.

**Results:**

In this diagnostic study of 64 patients with both PEth and SCRAM measures over 4 weeks (54 [84.4%] men; mean age, 65.5 [95% CI, 62.6-68.5] years; 51 [79.7%] White), 31 (48.4%) had any SCRAM-detected heavy alcohol consumption over the 4 weeks, and the median (IQR) PEth at 4 weeks was 23 ng/mL (<limit of quantification to 60 ng/mL). The AUC for PEth vs any SCRAM-detected heavy alcohol consumption was 0.83 (95% CI, 0.72-0.93). The optimal PEth cutoff was 18.5 ng/mL (AUC, 0.83; 95% CI, 0.72-0.93), with sensitivity of 83.9% (95% CI, 66.3%-94.5%) and specificity of 72.7% (95% CI, 54.5%-86.7%). The PEth test characteristics by individual drink reporting using the event monitors and by the AUDIT-C, and by these measures and by SCRAM collected for 2-week intervals, were similar to those compared with the 4-week SCRAM.

**Conclusions and Relevance:**

In a predominately middle-age and older White male population, PEth compared well with SCRAM. A PEth cutoff of 18.5 ng/mL (or rounded to 20 ng/mL, a recommended PEth cutoff for significant alcohol consumption) can be used in clinical care to detect heavy alcohol consumption in middle-age and older men.

## Key Points

**Question:**

What is the best cutoff for phosphatidylethanol (PEth), a blood biomarker, to objectively detect prior month heavy alcohol use?

**Findings:**

In this diagnostic study of 64 persons of middle age and older with paroxysmal atrial fibrillation, PEth was highly sensitive (84%) and specific (73%) for heavy alcohol use in the prior 4 weeks compared with readings from a continuous ankle monitoring to detect alcohol excreted in sweat. The optimal cutoff for heavy alcohol consumption was 18.5 ng/mL.

**Meaning:**

These findings suggest that PEth with this cutoff value (or rounded to the recommended cutoff of 20 ng/mL for significant alcohol consumption in the general population) could be used to accurately capture heavy alcohol use in middle-age and older men in clinical care.

## Teaser

This diagnostic study evaluates the optimal cutoff for phosphatidylethanol for heavy alcohol consumption in a study of middle-age and older adults.

## Introduction

Alcohol use increases the risk of communicable and several noncommunicable diseases, traumatic injury, and social harms, and it affects millions of people worldwide. It is the third leading cause of preventable death in the United States.^1^ Accurate assessment of heavy alcohol use, defined as the range of alcohol use that exceeds recommended drinking limits^2^ to alcohol use disorder, is urgently needed for clinical practice to determine the need for interventions and to inform medical care. While the US Preventative Services Task Force recommends screening and brief intervention for heavy alcohol use in primary care settings,^3^ many, but not all, patients (70%) reported they had been asked about alcohol use in the past year.^4^ Clinicians may be less likely to screen for alcohol use if they themselves drink regularly^5^ or due to discomfort, inadequate training, or judgmental attitudes.^6,7^ When clinicians do screen for alcohol use, self-report may suffer from a variety of biases, including social desirability bias and recall bias. Thus, while self-report can provide important information, it is key to develop additional methods to identify heavy alcohol consumption in clinical settings. This is especially salient as the US population ages, becomes more susceptible to alcohol’s effects,^8^ and experiences age-related multimorbidity, including frailty and cognitive decline, and increased prescription medicine–taking that may interact with alcohol consumption.

Biomarkers of alcohol use can be useful in clinical practice as objective measures that can overcome gaps in screening and inaccurate self-report in primary care. Furthermore, alcohol biomarkers can also be warning signs of risk for alcohol use disorder and tools for determining the etiology of health conditions, such as liver disease, and setting quantifiable goals for change.

Phosphatidylethanol (PEth) is an abnormal phospholipid that is formed only in the presence of ethanol and is therefore highly specific and highly sensitive.^9,10^ It can be detected in whole blood and dried blood spots (DBS). Several alcohol administration experiments have detected PEth formation soon after alcohol consumption and observed declines after alcohol consumption stopped.^11,12,13^ PEth is correlated with the volume of alcohol consumed in the prior month, with correlations between 0.57 and 0.69.^14^ The lack of a stronger correlation is due to interindividual variability in ethanol metobolism,^11^ and PEth may differ by hemoglobin levels, liver fibrosis, and body mass index.^15^ Despite this, PEth has shown very high sensitivity (94.5%-99%) and specificity (100%) for high alcohol consumption,^16,17^ and good sensitivity for lower levels of alcohol consumption (81.8%)^15^ with area under the curve (AUC, measuring predictive ability) for distinguishing drinking 1 to 2 glasses of wine per day from abstinence over 3 months of 0.92.^18^

For PEth to be useful to detect heavy alcohol use in clinical settings, appropriate cutoffs are needed.^10^ Some researchers have proposed a variety of PEth cutoffs for heavy alcohol consumption, ranging from 20 to 80 ng/mL; however, these cutoffs were derived from studies that relied on retrospective self-report of alcohol use, which themselves may be subject to recall bias and inaccurate reporting.^19,20,21^ Thus there is a need to derive PEth cutoffs most indicative of heavy alcohol consumption.

Our aim was to determine the optimal PEth cutoff for heavy alcohol use using an objective measure for comparison, ie, transdermal alcohol, detected in sweat via an ankle monitor worn for 4 weeks. This investigation was part of a study of the association between alcohol use and atrial fibrillation (AF), which was comprised of primarily middle-age and older White men.^22^ We compared these results with those obtained using 2 alternative measures of alcohol use, ie, from event-level self-reporting by pressing a button on a cardiac monitor when drinking alcohol and retrospective self-report.

## Methods

This analysis was part of a study to examine the association between alcohol consumption and AF.^22^ This study was approved by the institutional review board of the University of California, San Francisco. All participants gave written, informed consent to participate. To the extent possible, we followed the Standards for Reporting Diagnostic Accuracy (STARD) guidelines for reporting diagnostic studies.^23^

### Study Participants

Participants were recruited from the general cardiology and cardiac electrophysiology outpatient clinics for the parent study, from September 2014 to September 2019. Eligibility criteria for the original study included being age 21 years or older, diagnosed with paroxysmal AF and/or atrial flutter, and reporting consuming alcohol at least once a month; eligible patients were enrolled consecutively. Exclusion criteria were having a history of substance use disorder, scoring greater than 19 on the Alcohol Use Disorders Identification Test (AUDIT; to not appear to be condoning high-risk alcohol use), having planned changes in AF treatment (to best detect AF), having sensitivity to study procedural materials (eg, latex or adhesives), and being unwilling to wear an alcohol sensor for 4 weeks. The study included baseline, 2-week, and 4-week visits.

### PEth

Blood samples were collected via finger sticks to prepare DBS cards at the 2- and 4-week visits. The DBS cards were tested at the United States Drug Testing Laboratories using previously published methods.^24^ The testing measured the level of the 16:0/18:1 PEth analog; the limit of detection was 2 ng/mL and the limit of quantification (LOQ) was 8 ng/mL.

### Secure Continuous Remote Alcohol Monitor

Participants were asked to wear the Secure Continuous Remote Alcohol Monitor (SCRAM), a transdermal electrochemical ethanol sensor, around 1 ankle for 4 weeks. The SCRAM samples the air on the skin every 30 minutes. A systematic review of 19 studies that evaluated the use of SCRAM in clinical and nonclinical settings found moderate to high correlations between the transdermal alcohol measurement and self-report (*r* = 0.68-0.95) and estimated blood or breath alcohol concentrations (BrAC; *r* = 0.56-0.99), low malfunction rates (2%), 98.6% sensitivity, and 95.0% specificity for detecting 1 or more standard drink.^25^

### Alcohol Event Level Monitoring

Participants were issued an external remote cardiac event monitor, either the Lifewatch Ambulatory Cardiac Telemetry (ACT) Monitor (the first 37 participants) or the Zio Patch (iRythm; the remaining 63 participants), to wear during the 4 weeks of the study. We switched to the Zio Patch due to its improved ease of wear (a sticky patch vs electrodes). These monitors automatically detect AF independent of heart rate and include a patient activator button, which participants were instructed to press when they took a drink of alcohol, defined as a 12-ounce can or bottle of beer, a glass of wine, or a 1.5 ounce shot of spirits.

### Study Interviews

Participants completed interviewer-administered questionnaires at each visit. The interviews included the AUDIT-Consumption (AUDIT-C) questionnaire.^26^ The AUDIT-C contains 3 items measuring frequency of drinking, typical number of drinks, and heavy episodic drinking and was administered in modified version to represent only the prior 2 and 4 weeks at the 2- and 4-week visits, respectively. Race and ethnicity and biologic sex were considered relevant to incident AF^27^; race and ethnicity was elicited from participants using predetermined categories (American Indian or Alaska Native, Asian, Black or African American, Latino [ethnicity], Native Hawaiian or Other Pacific Islander, White, and other [not further defined]). Participants were asked to identify as male or female without reference to biologic sex or gender.

### Heavy Alcohol Consumption Definitions

We created variables to approximate the National Institute on Alcohol Abuse and Alcoholism heavy alcohol consumption definition, ie, more than 7 drinks per week or more than 3 drinks on any day for women and more than 14 drinks per week or more than 4 drinks on any day for men,^2^ using 3 measures (SCRAM, event monitor, and AUDIT-C). We included binge drinking, ie, consuming alcohol at a rate that brings one’s blood alcohol content to 0.08%, in this definition as well.

#### SCRAM Heavy Alcohol Consumption Definition

We used the Transdermal Alcohol Sensor Data Macro (TASMAC)^28^ to identify drinking episodes from the input SCRAM data. The TASMAC uses more liberal cutoffs of transdermal alcohol to indicate drinking episodes as compared with the SCRAM manufacturer’s calculations, which are considered overly conservative for research purposes, and also calculates estimated BrAC.^29^ Using the less conservative criteria, the SCRAM has high sensitivity (>89%) for detecting more than 2 drinks consumed per occasion by women and more than 3 drinks consumed per occasion by men.^29^ Thus, we defined heavy alcohol consumption in any week via SCRAM using 2 criteria: if SCRAM detected 3 or more (for women) and 4 or more (for men) drinking episodes or if the estimated BrAC for any drinking episode was 0.08% or greater. Heavy drinking via SCRAM was our primary reference standard, ie, the best available representation of truth.

#### Event Monitor Heavy Alcohol Consumption Definition

Participants were instructed to press the monitor button to record consumption of individual drinks. We defined heavy alcohol consumption as more than 7 presses per week or more than 3 presses on any day for women and more than 14 presses per week or more than 4 presses on any day for men.

#### AUDIT-C Heavy Alcohol Consumption Definition

Lastly, we considered AUDIT-C positive scores using established cutoffs for heavy alcohol consumption.^26^ The cutoff for women was 3 or greater; for men, it was 4 or greater.

### Statistical Analysis

We calculated descriptive statistics for the study sample at baseline for participants with complete PEth and SCRAM data at 4 weeks. We compared the characteristics of those with complete data at 4 weeks with those missing 4-week data by age, sex, race and ethnicity, and baseline AUDIT-C score (eTable 1 in Supplement 1). For descriptive purposes, we calculated Spearman correlations and 95% CIs (bootstrapped) for continuous versions of the alcohol measures (PEth in ng/mL, total number of SCRAM-positive episodes, total number of event monitor presses, and AUDIT-C score) at 4 weeks. Lastly, we constructed a receiving operator characteristic (ROC) curve and calculated the AUC and corresponding 95% CIs (bootstrapped) for PEth using heavy alcohol consumption determined by SCRAM. We next found the optimal PEth cutoff using the Youden J index^30^ (sensitivity plus specificity minus 1) which balances the need for high sensitivity with the need for high specificity. We calculated the sensitivity, specificity, positive predictive value (PPV), and negative predictive value (NPV) and 95% CIs for this cutoff. We chose the SCRAM measure as the primary comparator because it is a purely objective measure of alcohol use with high correlation with alcohol consumption.^25^ We conducted similar analyses using self-reported heavy alcohol use as measured by event monitor presses and by AUDIT-C, as defined previously, to determine the robustness of the findings to other metrics of alcohol use. Lastly, we conducted similar analyses using the data collected at the 2- and 4-week visits, using a 2-week look-back period, to represent alcohol use occurring over the prior 2 weeks, presented in eTables 2, 3, and 4 in Supplement 1. The analyses were conducted using Stata version 14 (StataCorp) using the corrci and diagt user written programs and were conducted from October 2021 to March 2022.

## Results

A total of 100 persons were enrolled in the original study. Of those, 64 had both PEth and SCRAM results at 4 weeks. The majority of these 64 persons were male (54 [84.4%]), and 10 (15.6%) were female; 6 (9.5%) were aged 21 to 49, 22 (34.9%) were aged 50 to 65, and 35 (55.6%) were older than 65 years; the mean age was 65.5 (95% CI, 62.6-68.5) years (Table 1). The race and ethnicity of participants was 3 (4.7%) African American, 8 (12.5%) Asian, 51 (79.7%) White, and 2 (3.1%) other (not specified). The 36 participants missing 4-week data did not differ from the 64 with 4-week data by age, sex, race and ethnicity, or baseline AUDIT-C score (eTable 1 in Supplement 1). The proportion meeting criteria for heavy alcohol consumption via SCRAM in any week in the prior 4 weeks was 48.4% (31 participants); the median (IQR) PEth at 4 weeks was 23 ng/mL (<LOQ to 60 ng/mL) (Table 2). The Spearman correlations of PEth with the number of SCRAM positive episodes in the prior 4 weeks was 0.66 (95% CI, 0.50-0.78) (Table 3). The AUC for PEth compared with heavy alcohol consumption in the prior 4 weeks measured by SCRAM was 0.83 (95% CI, 0.72-0.93) (Table 4), and the ROC curves for PEth vs heavy alcohol consumption measured by SCRAM, event monitoring, and AUDIT-C are shown in the Figure. The optimal cutoff for heavy alcohol consumption as measured by PEth was 18.5 ng/mL, which corresponded to a sensitivity of 83.9% (95% CI, 66.3%-94.5%) and a specificity of 72.7% (95% CI, 54.5%-86.7%). The PPV at 18.5 ng/mL was 74.3% (95% CI, 56.7%-87.5%), and the NPV was 82.8% (95% CI, 64.2%-94.2%). The AUCs for the other measures of 4-week alcohol consumption were in a similar range (all >0.79), and the corresponding optimal cutoffs ranged from 16.0 to 34.5 ng/mL (Table 4). The data analyzed by 2-week look-back intervals (at the 2- and 4-week visits) are described in eTables 2, 3, and 4 and the eFigure in Supplement 1. The correlations between PEth and the other measures using the 2-week look-back were lower than those for the 4-week period, while the AUC for PEth compared with heavy alcohol consumption detected by the SCRAM, looking back over 2 weeks, was similar (0.82; 95% CI, 0.74-0.90) to that for the 4-week period.

**Table 1.** Study Characteristics of 64 Participants at Baseline

Characteristic	Participants, No. (%)
Sex	
Male	54 (84.4)
Female	10 (15.6)
Age, mean (95% CI), y	65.5 (62.6-68.5)
Age, y	
21-49	6 (9.5)
50-65	22 (34.9)
>65	35 (55.6)
Race and ethnicity	
African American	3 (4.7)
Asian	8 (12.5)
White	51 (79.7)
Other^a^	2 (3.1)
AUDIT-C score, median (IQR)	4 (3-4)
Heavy alcohol consumption^b^	
Yes	37 (57.8)
No	27 (42.2)

Abbreviation: AUDIT-C, Alcohol Use Disorders Identification Test–Consumption.

^a^ Other race was not further specified.

^b^ Heavy alcohol consumption defined as an AUDIT-C score of 3 or greater for women and 4 or greater for men.

**Table 2.** Heavy Alcohol Consumption at 4-Week Follow-Up by Phosphatidylethanol (PEth), Secure Continuous Remote Alcohol Monitor (SCRAM), Event Monitor, and Self-Report Among 64 Participants

Measure	Participants, No./total No. (%)
PEth, median (IQR), ng/mL	23 (<LOQ to 60)
SCRAM heavy alcohol consumption^a^	31/64 (48.4)
Event monitoring heavy alcohol consumption^b^	20/63 (31.8)
AUDIT-C heavy alcohol consumption, modified or the prior 4 wk^c^	49/63 (77.8)

Abbreviations: AUDIT-C, Alcohol Use Disorders Identification Test–Consumption; LOQ, limit of quantification.

^a^ SCRAM heavy alcohol consumption defined as 3 or more (women) or 4 or more (men) SCRAM-detected drinking episodes or estimated breath alcohol content of 0.08% or greater during any week.

^b^ Event monitoring heavy alcohol consumption defined as more than 7 button presses per week or more than 3 on any day for women and more than 14 button presses per week or more than 4 on any day for men.

^c^ AUDIT-C heavy alcohol consumption defined as a score of 3 or greater for women and 4 or greater for men.

**Table 3.** Spearman Correlations Between Measures of Alcohol Consumption

Measure	ρ (95% CI)		
	PEth, ng/mL	SCRAM (total No. of SCRAM positive episodes)	Event monitoring (total No. of drinks)
PEth, ng/mL	1.00	NA	NA
Total No. of SCRAM-positive episodes	0.66 (0.50-0.78)	NA	NA
Total No. of drinks by event monitoring	0.73 (0.58-0.82)	0.54 (0.33-0.69)	NA
AUDIT-C score (0-12), modified for the prior 4 wk	0.74 (0.60-0.83)	0.54 (0.34-0.70)	0.72 (0.57-0.82)

Abbreviations: AUDIT-C, Alcohol Use Disorders Identification Test–Consumption; NA, not applicable; PEth, phosphatidylethanol; SCRAM, Secure Continuous Remote Alcohol Monitor.

**Table 4.** Phosphatidylethanol (PEth) Area Under the Receiver Operating Characteristic Curves (AUCs), Sensitivity, Specificity, and Cutoffs for Heavy Alcohol Consumption at Optimal Cutoff, Using the Youden J Statistic

Measure	AUC (95% CI)	Sensitivity, No./ total No. [% (95% CI)] at best cutoff	Specificity, No./total No. [% (95% CI)] at best cutoff	PEth cutoff at maximum Youden J, ng/mL
SCRAM heavy alcohol consumption^a^	0.83 (0.72-0.93)	26/31 [83.9 (66.3-94.5)]	24/33 [72.7 (54.5-86.7)]	18.5
Event monitor heavy alcohol consumption^b^	0.79 (0.67-0.90)	15/20 [75.0 (50.9-91.3)]	33/43 [76.7 (61.4-88.2)]	34.5
AUDIT-C heavy alcohol consumption^c^	0.90 (0.83-0.97)	37/49 [75.5 (61.1-86.7)]	14/14 [100.0 (76.8-100.0)]^d^	16.0

Abbreviations: AUDIT-C, Alcohol Use Disorders Identification Test–Consumption; SCRAM, Secure Continuous Remote Alcohol Monitor.

^a^ SCRAM heavy alcohol consumption defined as 3 or more (women) or 4 or more (men) SCRAM-detected drinking episodes or estimated breath alcohol content of 0.08% or greater during any week.

^b^ Event monitoring heavy alcohol consumption defined as more than 7 button presses per week or more than 3 on any day for women and more than 14 button presses per week or more than 4 on any day for men.

^c^ AUDIT-C heavy alcohol consumption defined as a score of 3 or greater for women and 4 or greater for men.

^d^ One-sided CI.

**Figure.**
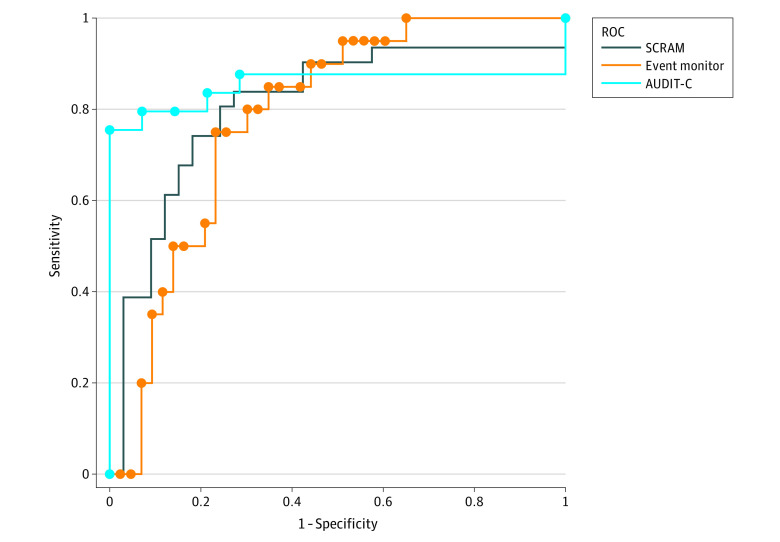
Receiver Operating Characteristic (ROC) Curve for Phosphatidylethanol vs Heavy Alcohol Consumption Measured by Secure Continuous Remote Alcohol Monitor (SCRAM), Event Monitoring, and Alcohol Use Disorders Identification Test–Consumption (AUDIT-C)

## Discussion

We found that the optimal PEth cutoff for heavy alcohol consumption was 18.5 ng/mL compared with real-time objective transdermal measurement via the SCRAM, and this cutoff was 83.9% sensitive and 72.7% specific for heavy alcohol use. This is the first study that we are aware of to examine PEth cutoffs for heavy alcohol consumption in comparison with a transdermal alcohol sensor worn over an extended period (4 weeks). PEth also had good sensitivity and specificity compared with real-time self-report via the event monitoring and self-reported heavy alcohol consumption via the AUDIT-C.

The optimal PEth cutoff of 18.5 ng/mL is lower than cutoffs proposed based on comparisons with self-reported alcohol use in a population-based study in Norway, which found that 40 ng/mL was the optimal cutoff for detecting drinking more than 1 drink per day, which would meet criteria for heavy alcohol consumption in women, and 61 ng/mL for detecting drinking more than 2 drinks per day, which would meet criteria for heavy alcohol consumption for men.^21^ A likely explanation of the lower PEth cutoffs for heavy alcohol consumption in our study is that the mean age in our study was 66 years and all participants had a history of AF, compared with a mean age of 55 years in volunteers in the study conducted in Norway.^21^ While age was not associated with the sensitivity of PEth for unhealthy alcohol use in an individual-participant data meta-analysis (with median age of 38),^15^ a recent study that purposefully enrolled persons over age 60 years found that PEth values were negatively correlated with age.^31^

Our optimal cutoff was quite near the proposed consensus cutoff for general populations of 20 ng/mL for detecting alcohol use greater than low consumption or abstinence^32^; this cutoff was also suggested in a review of the literature.^14^ However, in this study, PEth greater than 18.5 ng/mL indicated heavy alcohol consumption, as opposed to the suggested cutoff of 20 ng/mL for indicating greater than minimal alcohol consumption, which is a broader definition than heavy alcohol consumption. In our data, a cutoff of 20 ng/mL was 80.6% sensitive and 75.8% specific for heavy alcohol consumption (data not shown), suggesting rounding up to 20 ng/mL would be acceptable.

In populations similar to ours, eg, middle-age and older age men with heart conditions, 18.5 (or 20) ng/mL may indicate heavy alcohol consumption, and such patients could benefit from interventions to reduce alcohol use. We found that the Spearman correlations between PEth level and the number of SCRAM-detected drinking episodes, as well as between PEth level and the number of event monitor presses and between PEth level and the AUDIT-C score, were all greater than 0.66. These are good correlations, and consistent with the prior PEth literature^14^ and the knowledge that PEth is not perfectly correlated with alcohol consumption due to individual differences in alcohol metabolism.^11^ Lastly, our results suggest that PEth is more strongly correlated with 4-week than 2-week alcohol use.

### Limitations and Strengths

This study has limitations, including the modest sample size and the homogeneity by age, sex, and race and ethnicity of the study participants. As our cohort was comprised primarily of middle-age and older White men with AF, our findings should only be extrapolated to other populations with caution. Further work is needed to determine appropriate PEth cutoffs for younger populations and across sexes and racial groups, and to determine whether medications for AF and other conditions affect PEth levels. We also note that we excluded those with AUDIT scores greater than 19; thus, our cutoff is most relevant for heavy alcohol use that does not meet the level of alcohol use disorder. Another limitation is that the AUDIT-C was originally designed to assess prior year alcohol consumption; our modifications to assess the prior 2 and 4 weeks was new and has not been validated.

A strength of this study is the use of an objective real-time measure of alcohol use via a transdermal alcohol sensor (ie, the SCRAM) as the reference standard. However, we note that the SCRAM is less sensitive for single drink episodes compared with episodes with a greater number of drinks consumed^29^; thus, some alcohol consumption may have been misclassified. Despite this, the results were quite consistent across measures of heavy alcohol consumption, including those obtained by real-time self-report of individual alcohol-containing beverages consumed using event monitors and retrospective self-report using the AUDIT-C adapted to the appropriate time period. We note that the event monitor may have been susceptible to inadvertent button presses and retrospective self-report is often prone to bias. However, in this study, the number of drinks reported using the event monitor, the AUDIT-C, and the PEth results were well correlated. We suggest that this population, with a known symptomatic heart condition and self-reported alcohol consumption at study entry, was likely motivated by the study aims of understanding alcohol’s role in AF to accurately recall their alcohol use. Additionally, participants were made aware of how much alcohol they consumed because they were asked to report every alcohol-containing beverage using the event monitor; therefore, self-report was a good measure of alcohol consumption in this study.

## Conclusions

In conclusion, our findings suggest that PEth greater than 18.5 ng/mL is sensitive and specific for heavy alcohol consumption in the prior 4 weeks by middle-age and older men or populations with health conditions such as AF. More work is needed to determine optimal cutoffs in other populations. PEth levels greater than 18.5 ng/mL (or greater than the 20 ng/mL cutoff) in middle-age and older men can be used to indicate heavy alcohol consumption in clinical care that may need further intervention.

## Notes

Supplement 1. **eTable 1.** Comparison of Baseline Characteristics of Study Participants Included and Excluded From Analyses **eTable 2.** Heavy Alcohol Consumption Using a Prior 2-Week Look-Back Period by PEth, Transdermal Monitor (SCRAM), Event Monitor, and Self-Report (AUDIT-C), Among 140 Observations **eTable 3.** Spearman Correlations Between Measures of Alcohol Consumption Using a Prior 2-Week Look-Back Period, With 95% CIs, Among 140 Observations **eTable 4.** Phosphatidylethanol (PEth) AUCs, Sensitivity, Specificity and PEth Cutoffs for Heavy Alcohol Consumption Using a Prior 2-Week Look-Back Period at Optimal Cutoff Using the Youden J Statistic **eFigure.** ROC for PEth vs Heavy Alcohol Consumption Measured by SCRAM, Event Monitoring, and AUDIT-C: 2-Week Look-Back

Supplement 2.
Data Sharing Statement
