# Supplementary material for: Phosphatidylethanol vs Transdermal Alcohol Monitoring for Detecting Alcohol Consumption Among Adults
Source: JAMA Netw Open. 2023 Sep 12;6(9):e2333182. doi: 10.1001/jamanetworkopen.2023.33182 (PMC10498325; doi:10.1001/jamanetworkopen.2023.33182)
Supplement: Supplement 1. — eTable 1. Comparison of Baseline Characteristics of Study Participants Included and Excluded From Analyses eTable 2. Heavy Alcohol Consumption Using a Prior 2-Week Look-Back Period by PEth, Transdermal Monitor (SCRAM), Event Monitor, and Self-Report (AUDIT-C), Among 140 Observations eTable 3. Spearman Correlations Between Measures of Alcohol Consumption Using a Prior 2-Week Look-Back Period, With 95% CIs, Among 140 Observations eTable 4. Phosphatidylethanol (PEth) AUCs, Sensitivity, Specificity and PEth Cutoffs for Heavy Alcohol Consumption Using a Prior 2-Week Look-Back Period at Optimal Cutoff Using the Youden J Statistic eFigure. ROC for PEth vs Heavy Alcohol Consumption Measured by SCRAM, Event Monitoring, and AUDIT-C: 2-Week Look-Back [file jamanetwopen-e2333182-s001.pdf]

Supplemental Online Content

Hahn JA, Fatch R, Barnett NP, Marcus GM. Phosphatidylethanol vs transdermal alcohol monitoring for detecting alcohol consumption among adults. *JAMA Netw Open.* 2023;6(9):e2333182. doi:10.1001/jamanetworkopen.2023.33182

eTable 1. Comparison of Baseline Characteristics of Study Participants Included and Excluded From Analyses

eTable 2. Heavy Alcohol Consumption Using a Prior 2-Week Look-Back Period by PEth, Transdermal Monitor (SCRAM), Event Monitor, and Self-Report (AUDIT-C), Among 140 Observations

eTable 3. Spearman Correlations Between Measures of Alcohol Consumption Using a Prior 2-Week Look-Back Period, With 95% CIs, Among 140 Observations

eTable 4. Phosphatidylethanol (PEth) AUCs, Sensitivity, Specificity and PEth Cutoffs for Heavy Alcohol Consumption Using a Prior 2-Week Look-Back Period at Optimal Cutoff Using the Youden J Statistic

eFigure. ROC for PEth vs Heavy Alcohol Consumption Measured by SCRAM, Event Monitoring, and AUDIT-C: 2-Week Look-Back

This supplemental material has been provided by the authors to give readers additional information about their work.

eTable 1. Comparison of Baseline Characteristics of Study Participants Included and Excluded From Analyses				
	**All participants (n=100)**	**Excluded (n=36)**	**Included (n=64)**	**X2 or Mann-Whitney ranksum test (p-value)**
Gender, n (%)				3.10 (0.078)
Male	79 (79.0)	25 (69.4)	54 (84.4)	
Female	21 (21.0)	11 (30.6)	10 (15.6)	
Age, mean (95% confidence interval)	65.3 (63.0-67.6)	64.9 (61.0-68.7)	65.5 (62.6-68.5)	-0.17 (0.867)
Age, n (%)				0.45 (0.800)
21-49	9 (9.1)	3 (8.3)	6 (9.5)	
50-65	37 (37.4)	15 (41.7)	22 (34.9)	
>65	53 (53.5)	18 (50.0)	35 (55.6)	
Race/ethnicity, n (%)				4.71 (0.195)
African American	3 (3.0)	0 (0.0)	3 (4.7)	
Asian	9 (9.0)	1 (2.8)	8 (12.5)	
White	85 (85.0)	34 (94.4)	51 (79.7)	
Other	3 (3.0)	1 (2.8)	2 (3.1)	
AUDIT-C, median (IQR)	4 (3-4)	3 (3-4)	4 (3-4)	-0.61 (0.539)
Heavy alcohol consumption (AUDIT-C ≥ 3 for women, ≥ 4 for men), n (%)				0.24 (0.626)
Yes	56 (56.0)	19 (52.8)	37 (57.8)	
No	44 (44.0)	17 (47.2)	27 (42.2)	

© 2023 Hahn JA et al. *JAMA Network Open.*

**eTable 2. Heavy Alcohol Consumption Using a Prior 2-Week Look-Back Period by PEth, Transdermal Monitor (SCRAM), Event Monitor, and Self-Report (AUDIT-C), Among 140 Observations**	
PEth, median (IQR), ng/mL	22 (<LOQ*-53)
SCRAM heavy alcohol consumption: ≥3 (women) or ≥4 (men) SCRAM detected drinking episodes, OR estimated BrAC>0.08, any week, n/N (%)	51/140 (36.4)
Event monitoring heavy alcohol consumption: >7 button presses per week or >3 on any day (women), >14 button presses or >4 on any day (men), n/N (%)	28/138 (20.3)
AUDIT-C heavy alcohol consumption: AUDIT-C ≥3 (women) or AUDIT-C ≥4 (men), modified for the prior 2 weeks, n/N (%)	29/66 (43.9)
LOQ = limit of quantification	

© 2023 Hahn JA et al. *JAMA Network Open.*

**eTable 3. Spearman Correlations Between Measures of Alcohol Consumption Using a Prior 2-Week Look-Back Period, With 95% CIs, Among 140 Observations**			
	PEth (ng/mL)	SCRAM (total # of SCRAM positive episodes)	Event monitoring (total # of drinks)
PEth (ng/mL)	1.00	---	---
SCRAM (total # of SCRAM positive episodes)	0.58 (0.45-0.68)	---	---
Event monitoring (total # of drinks)	0.46 (0.32-0.59)	0.39 (0.23-0.53)	---
AUDIT-C score (0-12), modified for the prior 2 weeks	0.66 (0.50-0.78)	0.50 (0.29-0.66)	0.39 (0.15-0.58)

© 2023 Hahn JA et al. *JAMA Network Open.*

**eTable 4. Phosphatidylethanol (PEth) AUCs, sensitivity, Specificity and PEth Cutoffs for Heavy Alcohol Consumption Using a Prior 2-Week Look-Back Period at Optimal Cutoff Using the Youden J Statistic**				
Measure	AUC (95% CI)	Sensitivity n/N (%, 95% CI) at best cutoff	Specificity n/N (%, 95% CI) at best cutoff	PEth cutoff (ng/mL) at max Youden's J
SCRAM heavy alcohol consumption: ≥3 (women) or ≥4 (men) SCRAM detected drinking episodes, OR estimated BrAC>0.08, any week	0.82 (0.74-0.90)	42/51<br>82.4% (69.1-91.6)	66/89<br>74.2% (63.8-82.9)	26.5
Event monitor heavy alcohol consumption: >7 button presses per week or >3 on any day (women), >14 button presses of >4 on any day (men)	0.78 (0.69-0.87)	24/28<br>85.7% (67.3-96.0)	71/110<br>64.5% (54.9-73.4)	27.5
AUDIT-C heavy alcohol consumption: AUDIT-C ≥3 (women) or AUDIT-C ≥4 (men), modified for the prior 2 weeks	0.77 (0.65-0.89)	23/29<br>79.3% (60.3-92.0)	25/37<br>67.6% (50.2-82.0)	17.0

© 2023 Hahn JA et al. *JAMA Network Open.*

eFigure. ROC for PEth vs Heavy Alcohol Consumption Measured by SCRAM, Event Monitoring, and AUDIT-C: 2-Week Look-Back

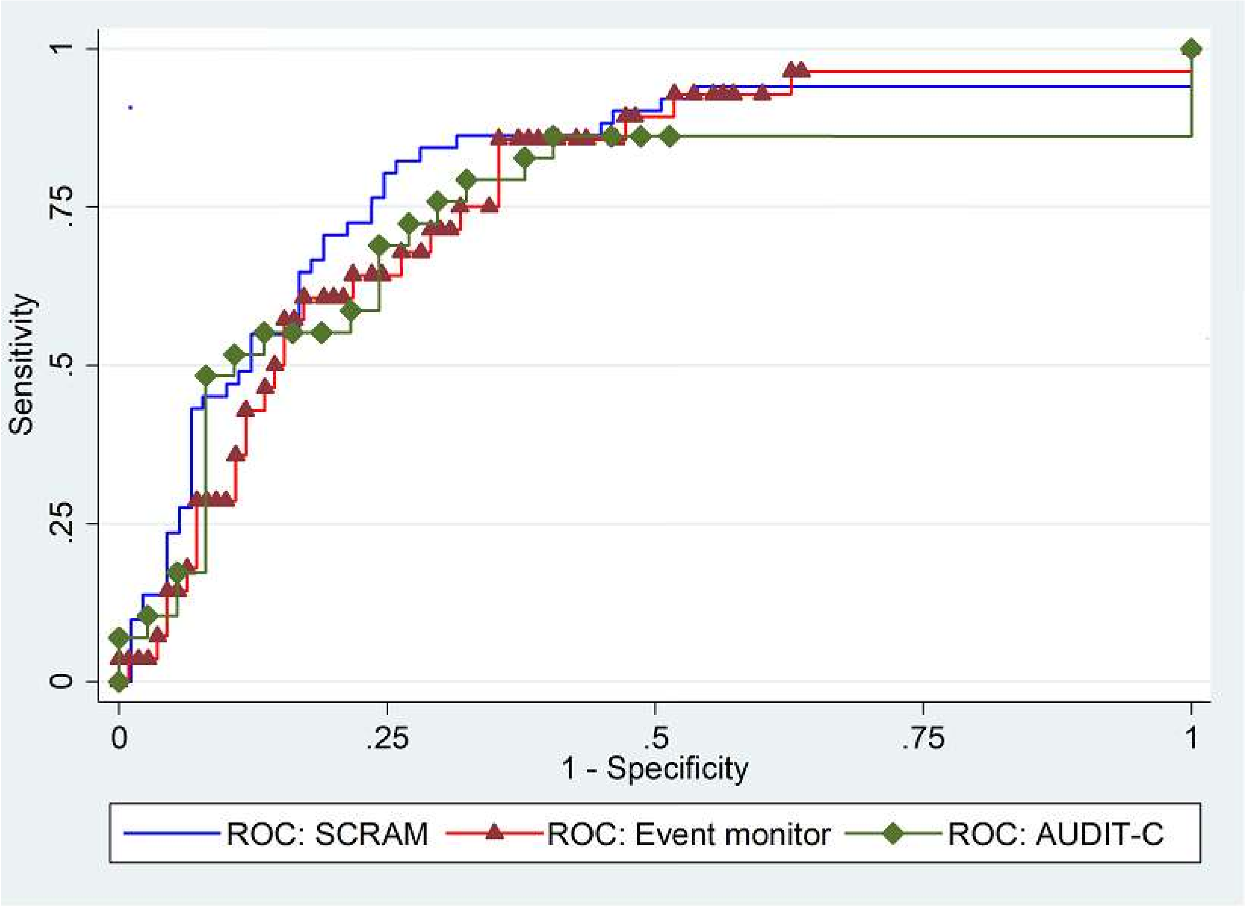

© 2023 Hahn JA et al. *JAMA Network Open.*
