# Supplementary material for: Phosphatidylethanol vs Transdermal Alcohol Monitoring for Detecting Alcohol Consumption Among Adults
Source: JAMA Netw Open. 2023 Sep 12;6(9):e2333182. doi: 10.1001/jamanetworkopen.2023.33182 (PMC10498325; doi:10.1001/jamanetworkopen.2023.33182)
Supplement: Supplement 2. — Data Sharing Statement [file jamanetwopen-e2333182-s002.pdf]

Data Sharing Statement

Hahn. Phosphatidylethanol vs Transdermal Alcohol Monitoring for Detecting Alcohol Consumption Among Adults. *JAMA Netw Open*. Published September 12, 2023. doi:10.1001/jamanetworkopen.2023.33182

Data
Data available: Yes
Data types: Deidentified participant data
How to access data: Data will be available upon request to robin.fatch@ucsf.edu
When available: With publication

Supporting Documents
Document types: None

Additional Information
Who can access the data: Researchers whose proposed use of the data has been approved.
Types of analyses: Any noncommercial purpose
Mechanisms of data availability: With a signed data access agreement
